# Antimicrobial Resistance and Predisposing Factors Associated with Catheter-Associated UTI Caused by Uropathogens Exhibiting Multidrug-Resistant Patterns: A 3-Year Retrospective Study at a Tertiary Hospital in Mogadishu, Somalia

**DOI:** 10.3390/tropicalmed7030042

**Published:** 2022-03-04

**Authors:** Abdikarim Hussein Mohamed, Nasteho Mohamed Sheikh Omar, Marian Muse Osman, Hussein Ali Mohamud, Aşır Eraslan, Metin Gur

**Affiliations:** Mogadishu Somalia Turkish Training and Research Hospital, Mogadishu 2526, Somalia; binasirshariif@gmail.com (N.M.S.O.); mariammo1994@gmail.com (M.M.O.); husseinalimohamud@gmail.com (H.A.M.); asireraslan@gmail.com (A.E.); drmetingur55@hotmail.com (M.G.)

**Keywords:** catheter-associated UTI, multidrug-resistant, intensive care unit

## Abstract

Background: Widespread and rapidly emerging multidrug-resistant uropathogens, particularly carbapenem-resistant pathogens, are a public health concern that impairs the determination of empirical therapy. This study aims to evaluate the antimicrobial susceptibility profile and factors associated with catheter-associated urinary tract infection (CA-UTI). Method: This retrospective study was carried out on a total of 779 urine cultures over a 3-year period. Antimicrobial sensitivity tests were performed using the standard Kirby–Bauer disk diffusion method. Results: The prevalence of CA-UTI in our study was 12.7%; a total of 47% of cultures had multi-drug-resistant (MDR) uropathogens, and 13% of the cultures showed extended-spectrum beta-lactamase (ESBL)-producing pathogens. Elderly patients, intensive care unit admissions, and associated comorbidities were correlated with higher rates of CA-UTI caused by multidrug-resistant uropathogens (*p* < 0.021, 95% CI: 0.893–2.010), (*p* < 0.008, 95% CI: 1.124–5.600), (*p* < 0.006, 95% CI: 0.953–2.617). Latex catheters and prolonged catheterization time were associated with increased risk of CA-UTI (*p* < 0.0001, 95% CI: 0.743–1.929, *p* = 0.012, 95% CI: 0.644–4.195). Patients with MDR uropathogens had prolonged hospital stays, i.e., 49% in more than 2 weeks (*p* < 0.04, 95% CI: 0.117–3.084). *E. coli* was the most common pathogen (26.3%), followed by *Acinetobacter baumannii* (24.3%). *Acinetobacter baumannii* showed the highest MDR pattern (88.5%), followed by *Pseudomonas aeruginosa* (68%). *Acinetobacter baumannii* and *Klebsiella pneumoniae* were associated with prolonged hospital stays (>2 w at 73.1 and 69%, respectively). Higher antimicrobial resistance against ceftriaxone (85.7%), meropenem (54.3%), ertapenem (50%), ciprofloxacin (58.5%), amikacin (27%), tigecycline (7.6%), and colistin (4.6%), was revealed in the study. Conclusion: Aside from the higher antimicrobial resistance against cephalosporins and fluoroquinolones, the findings of this study revealed that carbapenems are facing increased rates of antimicrobial resistance and are associated with substantial morbidity, prolonged hospitalization times, and increased healthcare expenses.

## 1. Introduction

Urinary tract infections (UTIs) are the most common infections worldwide and are a major healthcare obstacle that impacts over 150 million people annually [1]. Catheter-associated UTI (CA-UTI) is the most common hospital-acquired UTI, accounting for more than 80% of cases and affecting more than 1 million patients each year [2]. The risk of CA-UTI increases by 3–7% per day, as 20% of hospitalized patients are catheterized during admissions (45–79% of patients in intensive care units, as well as 17–23% of patients in medical and surgical wards) [3]. The term catheter-acquired infection refers to asymptomatic bacteriuria (CA-ASB), whereas CA-UTI refers to symptomatic patients [3]. CA-UTI is one of the most common nosocomial infections and leads to significant morbidity and increased healthcare expenditures, as it is associated with an increased number of multidrug-resistance (MDR) uropathogens with significant antimicrobial resistance [4,5]. More than 80% of isolated bacteria associated with CA-UTI are resistant to various classes of antimicrobials. 

Several studies have reported well-established risk factors, including the duration of catheterization, older age, female gender, urological procedures, and not maintaining a closed drainage system [6]. Many studies have reported that numerous uropathogens are resistant to commonly used antibiotics, which challenges the treatment and empiric therapy of patients with CA-UTI with MDR microorganisms [7]. Despite the increasing concern relating to antimicrobial-resistant pathogens, the number of UTI patients is increasing swiftly, and the nature of the infection is diverse according to local and geographical locations [8]. 

Increasing antimicrobial resistance rates among CA-UTI caused by uropathogens exhibiting MDR patterns is a worldwide concern. To date, there have been no studies reported from Somalia. This is the first study aiming to evaluate the prevalence of CA-UTI, the most common bacterial uropathogens, the length of hospital stay, factors and comorbidities associated with CA-UTI, and the antimicrobial susceptibility profile against uropathogens including MDR.

## 2. Method

This is a retrospective study conducted at Mogadishu Somali Turkish Reccep Tayyip Erdogan Training and Research Hospital over a 3 year period (from January 2019 to December 2021). This study included 99 patients with confirmed catheter-associated urinary tract infection whose urine cultures showed bacterial growth. The Centers for Disease Control and Prevention (CDC) defines CA-UTI as a symptomatic UTI episode following urinary catheterization for more than 48 h at the time of diagnosis with a single uropathogen with >100,000 CFU/mL in one urine culture [4].

All patients with newly inserted urethral catheters who developed CA-UTI in all ages and gender groups with symptoms of infection and catheter in situ for more than 48 h whose urine cultures showed bacterial growth were included in the study. Non-catheterized patients, those with positive urine culture within less than 48 h after admission or antimicrobial use prior to catheterization, were excluded from the study.

The analytic parameters include age, gender, side of admission (intensive care unit, surgical and medical wards), most common bacterial uropathogens, length of hospital stay, factors and comorbidities associated with CA-UTI, and the antimicrobial susceptibility profile against uropathogens including MDR. Antimicrobial resistance to at least one antibiotic drug in three or more antimicrobial classes (extended-spectrum penicillins, cephalosporins, fluoroquinolones, carbapenems, and aminoglycosides) was considered a multidrug-resistant uropathogen.

The catheter urine samples were aseptically obtained from the sampling port in the drainage system with a sterile needle after cleansing the sampling port of the urethral catheter. Samples were collected in well preserved containers and transferred promptly to the microbiology and laboratory unit, and were cultured on the selected medium cultures. The identification of the uropathogens was achieved by a combination of a BD BBL crystal identification system with a series of biochemical tests according to the system of the Clinical and Laboratory Standards Institute (CLSI). The growth of a single uropathogen with >100,000 CFU/mL represents CA-UTI [8]. The production of extended-spectrum beta-lactamases (ESBL) was identified from positive urine cultures using cephalosporins (ceftriaxone (30 µg), +cefotaxime (30 µg), +ceftazidime (30 µg)) and amoxicillin–clavulanic acid (20/10 µg) as the identifying disc to differentiate the patterns of growth between antibiotics with a lactamase inhibitor and those without. Subsequently, antimicrobial sensitivity tests were performed using the standard Kirby–Bauer disk diffusion method and commercial disks (Oxoid discs). Antimicrobial susceptibility testing (AST) against uropathogens was determined by the disk diffusion zone of growth inhibition and MIC values (susceptible (S) and resistant (R) categories) according to the CLSI criteria, except for breakpoints for tigecycline, where we used the FDA’s criteria for minimum inhibitory concentration (MICs) breakpoints for susceptible (≤2 mg/L), intermediate (4 mg/L), and resistant (≥8 mg/L) categories [9]. The minimum inhibitory concentration of 2 μg/mL colistin was the clinical breakpoint following the Clinical and Laboratory Standards Institute and European Committee on Antimicrobial Susceptibility Testing [10]. The studied antibiotics against pathogens were ciprofloxacin (5 Mcg), nitrofurantoin (300 Mcg), trimethoprim/sulfamethoxazole (1.25/23.75 Mcg), cephazolin (30 µg), meropenem (10 Mcg), ertapenem (10 Mcg), piperacillin (100 µg), piperacillin/tazobactam (100/10 Mcg), colistin (10 Mcg), linezolid (30 Mcg), amikacin (30 Mcg), tigecycline (15 µg), cefepime (30 µg), ceftazidime (30 µg), clindamycin (2 Mcg), penicillin (G 1 U), vancomycin (30 Mcg), daptomycin (30 Mcg), cefoxitin (30 Mcg), tetracycline (30 Mcg), erythromycin (15 Mcg), and teicoplanin (30 µg). 

The ethical committee of our institution received the ethics approval form (REF. MSTH-9005). Because we used the electronic medical records in the hospital information system and there was no harm to the patients, informed consent was not required. The descriptive univariate study design was used to analyze the analytic parameters. The frequencies and proportions were presented as point estimates in categorical variables, and the mean (±SD) in quantitative variables. To detect the significant association between the variables, the bivariate analysis was used. A *p* value of <0.05 was considered statistically significant. The binary logistic regression model included variables that exhibited statistical significance in the bivariate analysis. The 95% CIs were calculated to determine the association. All statistical analyses were performed using the Statistical Package for Social Sciences (SPSS-IBM) for Windows Version 23.

## 3. Results

This retrospective study was carried out on a total of 779 urine cultures performed in the microbiology unit of our hospital over a 3-year period. The prevalence of CA-UTI in our study was 12.7%. The mean age of the patients was 41.2 years. Elderly patients, intensive care unit admissions, and associated comorbidities were correlated with higher rates of CA-UTI caused by multidrug-resistant uropathogens (*p* < 0.021, 95% CI: 0.893–2.010), (*p* < 0.008, 95% CI: 1.124–5.600), (*p* < 0.006, 95% CI: 0.953–2.617). Latex catheters and prolonged catheterization time were associated with increased risk of CA-UTI, (*p* < 0.0001, 95% CI: 0.743–1.929, *p* = 0.012, 95% CI: 0.644–4.195). The mean length of the patients’ hospital stays was 20.9 days. There was no statistically significant difference in terms of length of hospital stay in ICU and non-ICU patients (*p* = 0.377). Patients with MDR uropathogens had prolonged hospital stays, with 49% staying at the hospital for more than 2 weeks (*p* < 0.04, 95% CI: 0.117–3.084). Sixty-four percent of the CA-UTI patients were male, and 36.4% were female. There was no statistically significant association between CA-UTI and gender groups (*p* = 0.061). Regarding the age distribution of the cases, the most predominant age group was 19–39 years (about 38.1%), followed by ≥60 years (about 29.3%), 40–59 years (23.75%), and the least predominant age group was under 18 years (about 10.3%). Most of the study cases were admitted in the ICU (58.5%), and 41.4% were admitted in the surgical and medical units (Table 1).

According to the distribution of uropathogens, *E. coli* was the most common pathogen (26.3%), followed by *Acinetobacter baumannii* (24.3%), *Pseudomonas aeruginosa* (19.2%), and *Klebsiella pneumoniae* (13.1%). Gram-negative uropathogens constituted 88.9% and Gram-positive constituted 11.1% of the total pathogens. *Acinetobacter baumannii* showed the highest MDR pattern (88.5%) and was the most predominant pathogen in the ICU (73.1), followed by *Pseudomonas aeruginosa* (68%). Forty-seven percent of the cases had multidrug-resistant (MDR) uropathogens. *Acinetobacter baumannii* and *Klebsiella pneumoniae* were associated with prolonged hospital stays (>2 weeks at 73.1% and 69%, respectively). Thirteen percent of the cultures showed ESBL-producing pathogens, and *Klebsiella pneumoniae* was the most predominant pathogen in 7/13 cases followed by *E. coli* (16.6%) (Table 2). Most of the ESBL-producing pathogens had an MDR pattern (about 61.5%, or 8/13).

The antimicrobial resistance pattern against the uropathogens is shown in Table 3. The highest antimicrobial resistance against uropathogens was associated with cephazolin (96%), cefuroxime (93.5%), ampicillin (94.3%), ceftriaxone (85%), and amoxicillin–clavulanic acid (75%). Higher antimicrobial resistance against carbapenems was revealed through the study, with 54.3% against meropenem, 50% in ertapenem, and 35% toward imipenem. We revealed an increasing antimicrobial resistance toward tigecycline (7.6%), colistin (4.6%), and vancomycin (14.3%). Flouroquinolones (ciprofloxacin at 58.5% and levofloxacin at 42.8%), and aminoglycosides (gentamycin at 59.3% and amikacin at 27%) were shown to be resistant to the pathogens. The study showed that more than half of *E. coli* isolates (59.5%) were resistant to fluoroquinolones (ciprofloxacin at 68.7% and levofloxacin at 52.4%), 61.6% of cephalosporins, and 7–30% of the carbapenems. Increasing antimicrobial resistance against *Pseudomonas aeruginosa* was noticed throughout the study in the following drugs: ceftazidime (53.8%), piperacillin–tazobactam and ciprofloxacin (14.3%), and colistin (14%). The lowest resistance rate against *Acinetobacter baumannii* was shown in tigecycline (0%), and colistin (4%). *Staphylococcus aureus* showed an antimicrobial resistance rate of about 16.6% against vancomycin and linezolid.

## 4. Discussion

Widespread and rapidly emerging multidrug-resistant (MDR) uropathogens, particularly carbapenem-resistant pathogens, are a public health concern that impairs the determination of empirical antibiotic therapy [11]. Carbapenem-resistant Enterobacteriaceae (CRE) isolates have grown in number in the last decades. This is a healthcare challenge requiring complex and prolonged antimicrobial management due to the associated morbidity and mortality despite the related MDR and XDR pattern. A prospective study of a total of 105 urine cultures from catheterized patients admitted to the intensive care unit reported that Gram-negative MDR uropathogens had higher antimicrobial resistance against meropenem (53.84%), imipenem (19.23%), and amikacin (46.15%), which coheres with our findings (35–54% antimicrobial resistance against carbapenems) [12]. Jayakaran, et al. reported that Gram-negative MDR uropathogens exhibited 37.6% antimicrobial resistance against carbapenems, mainly in ICU patients, which corresponds to the current study [13]. Knowledge about the local antimicrobial resistance rates, surveillance, and better adherence to the guidelines for improved diagnosis and management, and the future development of new antibiotic agents, are essential for the consideration of an optimal treatment of the MRD uropathogens.

The study showed that more than half of *E. coli* isolates (59.5%) were resistant to fluoroquinolones (ciprofloxacin in 68.7% and levofloxacin in 52.4%) and 61.6% of cephalosporins, which matches a consensus review in the Asia-Pacific region on the increased rates of antimicrobial resistance; this review reported that nearly half of *Escherichia coli* urinary isolates were resistant to fluoroquinolones and cephalosporins [14]. *E.coli* displayed high rates of antimicrobial resistance against fluoroquinolones (71%), which is higher than our findings [15]. *Acinetobacter baumannii* was the most frequent pathogen that exhibited an MDR and XDR pattern. Fortunately, the pathogens revealed a satisfiable sensitivity rate against tigecycline (100%) and colistin (96%). *Klebsiella pneumoniae*-related catheter-associated urinary tract infections were related to a higher rate of extended-spectrum β-lactamase (ESBL) expression (54%) and multidrug resistance (MDR) pattern (46%). *Klebsiella pneumoniae* were associated with prolonged hospital stays >2 weeks (69%) [16].

*Pseudomonas aeruginosa* is responsible for 12% of all catheter-associated UTIs, making it the third most common organism isolated from CA-UTI patients, as noticed in our study, after *E. coli* and *Acinetobacter baumannii* [17]. Increasing antimicrobial resistance against *Pseudomonas aeruginosa* was noticed throughout the study. A study of 40 patients with CA-UTI conducted by Barbadoro P and associates reported that the most common isolated pathogen was *Pseudomonas aeruginosa* (41.5%); all the isolated *Pseudomonas aeruginosa* were shown to be extensively drug resistant against medications, being susceptible only to colistin (100%) [6]. 

Elderly patients, latex catheters, prolonged catheterization time, and intensive care unit admissions were found to increase the likelihood of CA-UTI caused by MDR uropathogens [18]. A systematic review and meta-analysis of a total of 8785 UTI patients with and without catheters over 10 years by Li F, et al. reported that female gender, prolonged duration of catheterization and previous catheterization, diabetes, prolonged hospital stay, and intensive care unit admission led to a high risk for catheter-associated urinary tract infection in patients [19]. A total of 58.5% of our cases were admitted to the ICU, and ICU stay was significantly associated with the development of CA-UTI (odds ratio 1.122, 95% confidence interval 1.074–1.173, *p* < 0.001) as reported by Kim, B et al. [20]. 

The prevalence of CA-UTI in our study was 12.7%, which corresponds with that of previously reported studies [19]. Regarding the gender distribution of CA-UTI, our studies revealed that being male is a risk factor, with males accounting for 64.6% of cases, which agrees with the previously reported studies on this topic [19]. *E. coli* was the most common pathogen (26.3%), followed by *Acinetobacter baumannii* (24.3%), *Pseudomonas aeruginosa* (19.2%), and *Klebsiella pneumoniae* (13.1%). A systematic review including 75 five studies reported *Candida spp.* to be the most frequent pathogen isolated from CA-UTI patients (27.4%), followed by *Escherichia* spp. (23.41%) and *Enterococcus* spp. (15.0%) [15]. Similar to our findings, many studies have reported that *E. coli* followed by *Klebsiella pneumoniae*, was the most commonly isolated uropathogen from urine cultures [21]. In our study, 47% of cases had MDR uropathogens (antimicrobial resistance to at least one antibiotic drug in three or more antimicrobial classes). A cross-sectional study conducted in southern Ethiopia reported a higher rate of MDR isolates in 37 out of 42 patients (88.1%) [22]. CA-UTI is associated with a prolonged length of hospital stay (extra 900,000 days per year), increased comorbidity, and increased mortality rates (estimated 7500 deaths annually) [17].

The constant monitoring and reporting of local antimicrobial resistance patterns is crucial to help clinicians in the treatment and management of CAUTIs caused by uropathogens exhibiting multidrug-resistant patterns.

Strengths and limitations: This is a retrospective study missing some important factors that impact the development of CA-UTI caused by uropathogens exhibiting MDR patterns, including previous catheterization and previous antimicrobial use. Although this study is the first study reported from Somalia, it is a single-center-based study and the sample size is small. Continuous surveillance of the local antimicrobial resistance pattern is essential for the identification of an optimal treatment.

## 5. Conclusions

Increasing antimicrobial resistance rates among CA-UTI caused by uropathogens exhibiting MDR patterns is a worldwide concern. Forty-seven percent of the cases had multidrug-resistant (MDR) uropathogens. *Acinetobacter baumannii* showed the highest MDR patterns. Fortunately, the lowest resistance rates were displayed against tigecycline and colistin. The findings of this study revealed that being elderly, intensive care unit admissions, latex catheters, and prolonged catheterization time were found to increase the likelihood of CA-UTI caused by MDR uropathogens. Widespread and rapidly emerging MDR uropathogens, particularly carbapenem-resistant pathogens, are a public health concern that impairs the determination of empirical antibiotic therapy. 

## Figures and Tables

**Table 1:** Factors associated with CA-UTI caused by MDR-uropathogens.

Factors	MDR		*p*-Value	95% CI
	Yes	No		
Age ≤18 y 19–39 y 40–59 y ≥60 y	1 17 12 16	9 20 11 11	*p* < 0.021	0.893–2.010
Gender Male Female	28 18	34 17	*p* = 0.061	0.643–1.263
Site of admission Non-ICU ICU	13 33	28 23	*p* < 0.008	1.124–5.600
Catheter Latex Silicon	37 9	39 14	*p* < 0.0001	0.743–1.929
Duration of catheterization 2–4 days 5–10 days >10 days	6 19 21	23 26 4	*p* = 0.012	0.644–4.195
Comorbidities Renal failure Diabetes Cancer	8 12 3	15 6 8	*p* < 0.006	0.953–2.617
Length of hospital stay (LOS) 1–7 days 8–14 days >2 weeks	4 13 29	5 16 30	*p* < 0.04	0.117–3.084

**Table 2:** Distribution of uropathogens that showed bacterial growth including ESBL and MDR pathogens.

Type of Microorganisms	No. Patients	Percentage
Gram-negative pathogens	88	88.9%
*E. coli* ESBL-producing *E. coli* MDR Yes No	26 4 5 21	26.3%
*Klebsiella pneumoniae* ESBL-producing *Klebsiella* MDR Yes No	13 7 6 7	13.1%
*Acinetobacter baumannii* MDR Yes No	24 21 3	24.3%
*Pseudomonas aeruginosa* MDR Yes No	19 9 10	19.2%
*Proteus mirabilis* MDR Yes No	3 1 2	3%
*Enterobacter* spp. ESBL-producing MDR Yes No	1 1 0 1	1%
*Pantoea agglomerans* ESBL-producing MDR Yes No	1 1 0 1	1%
*Citrobacter freundii* MDR Yes No	1 0 1	1%
Gram-positive pathogens	11	11.1%
*Staphylococcus aureus* MDR Yes No	9 2 7	9.1%
*Enterococcus* spp. MDR Yes No	2 0 2	2%
Total	99	100.0%

**Table 3:** The antimicrobial resistance pattern against the medications including specific antimicrobial resistance patterns against Gram-negative uropathogens.

Medications	Total/Resistant	*E. coli*	*Klebsiella pneumoniae*	*Pseudomonas aeruginosa*	*Acinetobacter baumannii*
Cephazolin	25 (96%)	85.7%	100%	100%	100%
Cefotaxime	24 (79.2%)	66.6%	100%	50%	100%
Cefoxitin	41 (70.7%)	50%	71.4%	75%	100%
Cefuroxime	46 (93.5%)	100%	85.7%	85.7%	100%
Ceftriaxone	7 (85.7%)	100%	100%		100%
Ceftazidime	26 (53.8%)			53.8%	50%
Cefepime	20 (85%)			81.8%	100%
Cefixime	20 (55%)	66%	60%	30%	100%
Ampicillin	53 (94.3%)	100%	100%	100%	100%
Cefoperazone–sulbactam	49 (30.6%)	6.6%	60%	0%	100%
Amoxicillin–clavulanic acid	40 (75%)	53.3%	85.7%	88.9%	100%
Gentamicin	54 (59.3%)	61.5%	71.4%	45.5%	100%
Amikacin	63 (27%)	0%	0%	20%	73.6%
SMX-TMP	53 (84.9%)	91%	77.7%	89%	100%
Ciprofloxacin	65 (58.5%)	68.7%	50%	14.3%	94.1%
Levofloxacin	28 (42.8)	52.4%		66.6%	100%
Nitrofurantoin	3 (100%)				
Fosfomycin	3 (33.3%)				
Imipenem	54 (35.3%)	7.1%	11.1%	15.4%	92.8%
Meropenem	46 (54.3%)	10%	57.1%	50%	93.7%
Ertapenem	34 (50%)	30%	37.5%	80%	85.7%
Linezolid	10 (40%)	8.3%			
Piperacillin	26 (69.2%)	20%	57%		100%
Piperacillin–tazobactam	37 (48.6%)	33%	25%	14.3%	100%
Vancomycin	7 (14,3%)				
Tigecycline	26 (7.6%)	0%	0%	10.5%	0%
Colistin	43 (4.6%)	0%	0%	14%	4%
Daptomycin	6 (33.3%)				
Clindamycin	8 (12.5%)				
Ampicillin/sulbactam	7 (14.3%)				

## Data Availability

Data are included in the manuscript.

## Abbreviations

AST: antimicrobial susceptibility testing; CA-UTI: catheter-associated urinary tract infection; CDC: Centers for Disease Control and Prevention; CLSI: Clinical and Laboratory Standards Institute; ESBL: extended-spectrum beta-lactamases; ICU: intensive care unit; LOS: length of hospital stay; MDR: multidrug-resistant; UTI: urinary tract infection; XDR: extensive drug-resistance.
